# A novel gene-diet interaction promotes organismal lifespan and host protection during infection via the mitochondrial UPR

**DOI:** 10.1371/journal.pgen.1009234

**Published:** 2020-12-18

**Authors:** Mustafi Raisa Amin, Siraje Arif Mahmud, Jonathan L. Dowgielewicz, Madhab Sapkota, Mark W. Pellegrino

**Affiliations:** Department of Biology, University of Texas Arlington, Arlington, Texas, United States of America; Princeton, UNITED STATES

## Abstract

Cells use a variety of mechanisms to maintain optimal mitochondrial function including the mitochondrial unfolded protein response (UPR^mt^). The UPR^mt^ mitigates mitochondrial dysfunction by differentially regulating mitoprotective gene expression through the transcription factor ATFS-1. Since UPR^mt^ activation is commensurate with organismal benefits such as extended lifespan and host protection during infection, we sought to identify pathways that promote its stimulation. Using unbiased forward genetics screening, we isolated novel mutant alleles that could activate the UPR^mt^. Interestingly, we identified one reduction of function mutant allele (*osa3*) in the mitochondrial ribosomal gene *mrpl-2* that activated the UPR^mt^ in a diet-dependent manner. We find that *mrpl-2(osa3)* mutants lived longer and survived better during pathogen infection depending on the diet they were fed. A diet containing low levels of vitamin B12 could activate the UPR^mt^ in *mrpl-2(osa3)* animals. Also, we find that the vitamin B12-dependent enzyme methionine synthase intersects with *mrpl-2(osa3)* to activate the UPR^mt^ and confer animal lifespan extension at the level of ATFS-1. Thus, we present a novel gene-diet pairing that promotes animal longevity that is mediated by the UPR^mt^.

## Author summary

The mitochondrial unfolded protein response (UPR^mt^) is a cellular pathway that mediates recovery of damaged mitochondria. Mitigation of mitochondrial dysfunction is critical due to the essential nature of the organelle. The UPR^mt^ controls the expression of genes that help restore mitochondrial homeostasis via the bZIP transcription factor ATFS-1. The activation of the UPR^mt^ is associated not only with extended animal lifespan but also with increased host protection during infection with bacterial pathogens. Using a genetic screen to identify pathways that activate the UPR^mt^, we recovered a novel reduction of function mutation in the mitochondrial ribosomal gene *mrpl-2* that mediates protein translation in mitochondria. Interestingly, our isolated *mrpl-2* mutant activates the UPR^mt^ and promotes longevity when fed a specific diet which is low in the metabolite vitamin B12. We find that impaired function of methionine synthase, an enzyme that uses vitamin B12 as a cofactor to convert homocysteine to methionine, genetically interacts with the isolated *mrpl-2* mutant to activate the UPR^mt^ and extend lifespan. Our findings suggest that the lifespan-extending benefits of impaired mitochondrial translation and that of methionine restriction occur through a common mechanism that includes the activation of the UPR^mt^.

## Introduction

Because of their endosymbiotic origin, mitochondria possess their own genome and ribosomes that are used to express and translate a minor portion of the mitochondrial proteome [1]. The mitochondrial genome encodes 13 (12 in *C*. *elegans*) subunits of the multimeric electron transport chain (ETC) complexes while the remaining components are expressed from the nuclear genome, translated on cytosolic ribosomes and imported into mitochondria using a sophisticated import pathway [2]. This requires a great deal of coordination between both the mitochondrial and nuclear genomes in order to efficiently assemble these multimeric ETC structures. Imbalances in mitonuclear coordination causes mitochondrial dysfunction that is sensed by cellular defense programs to help restore normal organelle function [3–5]. Retrograde signaling such as the mitochondrial unfolded protein response (UPR^mt^) is one type of defense mechanism that is used to mitigate mitochondrial dysfunction [6,7]. Here, decreased mitochondrial function is coupled to changes in gene expression that helps restore mitochondrial homeostasis. At the center of the UPR^mt^ is the bZIP transcription factor ATFS-1 that coordinates the changes in gene expression associated with this mitochondrial stress response [8,9]. A defining characteristic of ATFS-1 is the presence of a mitochondrial targeting sequence that mediates its import into healthy mitochondria where it subsequently undergoes proteolytic degradation. Import efficiency is reduced in dysfunctional mitochondria allowing ATFS-1 to accumulate in the cytosol and be imported into the nucleus. ATFS-1 regulates a broad change in gene expression that in turn mediates mitochondrial recovery with roles in proteostasis, detoxification, and metabolic reprogramming [9].

Paradoxically, while a decline in mitochondrial function is associated with organismal aging and disease, there is considerable support that mild impairment can extend lifespan [10]. Conditions that reduce mitochondrial function and extend lifespan are also associated with the activation of the UPR^mt^. Evidence also exists demonstrating a requirement of the UPR^mt^ for mitochondrial stress-induced longevity. For example, the UPR^mt^ is required for the increase in lifespan that is observed in mitochondrial stressed animals with impaired ETC function [11,12]. Also, disruptions to mitochondrial ribosome function results in mitonuclear imbalances that extend lifespan in a UPR^mt^-dependent manner [3]. It is important to note that although UPR^mt^ activation is correlated with conditions that promote lifespan extension, it is not an absolute predictor of this phenomenon nor is it always required [5].

In addition to promoting lifespan extension, UPR^mt^ activation is also associated with promoting host survival during infection. Here, ATFS-1 regulates the expression of genes related to innate immunity including anti-microbial peptides, lysozymes and C-type lectins [9,13]. Consistently, ATFS-1 is required for protection during infection with pathogens that target mitochondrial function [13–15]. Also, priming the host for the UPR^mt^ prior to infection significantly improves host resistance [13,16].

Here, we have identified a reduction of function allele in the *C*. *elegans* mitochondrial ribosome gene *mrpl-2* using a forward genetics approach. We find that the *mrpl-2* mutant exhibits extended lifespan and increased survival during pathogen infection. However, the benefits conferred by *mrpl-2(osa3)* were diet-dependent. We find that a diet low in vitamin B12 acts synergistically with the *mrpl-2* mutant genetic background. Mechanistically, loss of the vitamin B12-dependent enzyme methionine synthase interacts with the *mrpl-2* mutant to drive the activation of the UPR^mt^, thus promoting lifespan extension. Our data support a model in which genetically-induced mitonuclear imbalance and diet-mediated methionine restriction use a common mechanism to promote lifespan extension, including the activation of the UPR^mt^.

## Results

### Forward genetic mutagenesis screen identifies novel alleles that activate the UPR^mt^ in diet-dependent and independent manners

**Fig 1:**
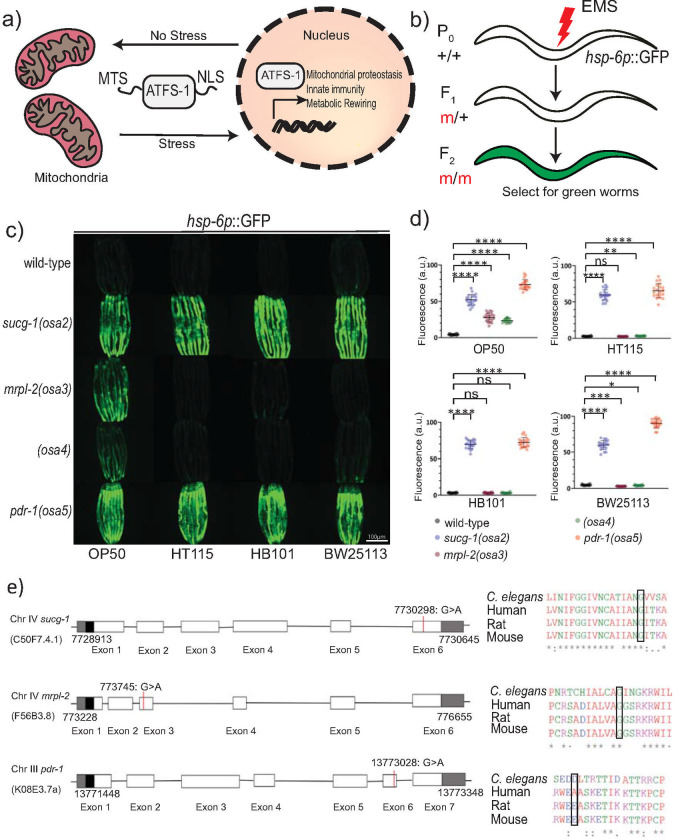
Isolation of novel alleles that activate the UPR^mt^ in diet-dependent and–independent manners. (A) Schematic of the UPR^mt^ response. (B) Schematic illustrating the strategy used to isolate mutants that activate the UPR^mt^ using forward genetics. (C, D) Photomicrographs and quantification of *hsp-6p*::GFP expression for the four isolated mutants fed different diets of *E*. *coli*. The alleles were named *osa2*, *osa3*, *osa4* and *osa5*. Quantification of fluorescence intensities expressed as arbitrary units (A.U.); (mean ±SD; *n* ≥ 20 worms); ns denotes not significant, * denotes *p*<0.05, ** denotes p ≤ 0.01, *** denotes p ≤ 0.001, **** denotes p ≤ 0.0001 (Student’s *t* test). (E) Schematics of gene structure and protein alignment of *sucg-1(osa2)*, *mrpl-2(osa3)*, and *pdr-1(osa5)* with the indicated mutations and amino acid changes.

Because activation of the UPR^mt^ is correlated with extended lifespan and increased survival during pathogen infection, we sought to perform a forward genetic screen to uncover novel alleles capable of activating the UPR^mt^ using the strain SJ4100 *hsp-6p*::GFP (Fig 1A and 1B). *C*. *elegans hsp-6* encodes the ortholog of the mitochondrial chaperone mtHSP70 and its expression is induced during the UPR^mt^ [17]. We obtained four independent viable mutants from this screen that could activate the *hsp-6p*::GFP reporter (mutant alleles *osa2-osa5*) (Fig 1C and 1D).

ATFS-1 is required for the development and/or fertility of mitochondrial stressed animals [9,18], prompting us to investigate the effect of its knockdown by RNAi in all our identified mutants. Interestingly, while loss of ATFS-1 slowed the development and reduced the fertility of *osa2* and *osa5* animals, it had negligible effects for *osa3* and *osa4* (S1A Fig). We therefore examined UPR^mt^ activity in *osa2*-*osa5* animals in the presence or absence of ATFS-1. Consistent with causing a delay in animal development, loss of ATFS-1 suppressed the induction of *hsp-6p*::GFP in *osa2* and *osa5* animals (S1B Fig). Surprisingly, *hsp-6p*::GFP expression was not induced in *osa3* and *osa4* animals even when grown with empty RNAi plasmid control bacteria (S1B Fig). RNAi by feeding in *C*. *elegans* uses the RNase III-deficient *E*. *coli* strain HT115 (*E*. *coli* K12-type strain) as opposed to the standard *E*. *coli* uracil auxotroph strain OP50 (*E*. *coli* B-type strain). Since we had performed the forward genetics mutagenesis using *E*. *coli* OP50, we hypothesized that the type of diet may be influencing the activation of the UPR^mt^ in *osa3* and *osa4* animals. Indeed, we could recapitulate the absence of UPR^mt^ activation in *osa3* and *osa4* animals using *E*. *coli* HT115 bacteria that lacked the empty RNAi plasmid (Fig 1C and 1D). We also tested another *E*. *coli* K12 strain BW25113 and the K12/B-type hybrid strain HB101 which similarly did not induce *hsp-6p*::GFP expression in *osa3* and *osa4* animals (Fig 1C and 1D). In contrast, the type of *E*. *coli* diet had no discernible effect on the activation of the UPR^mt^ in the *osa2* or *osa5* mutant backgrounds (Fig 1C and 1D). Thus, the type of diet influences the activation of the UPR^mt^ in the *osa3* and *osa4* genetic backgrounds.

We used whole genome sequencing to identify the genes responsible for the UPR^mt^ induction observed in *osa2-osa5*. The allele *osa2* contained a mutation (GGT➔AGT[359 Gly➔Ser] in *sucg-1*, the *C*. *elegans* homolog of SUCLG2 succinyl-CoA ligase subunit beta (Fig 1E). The allele *osa3* contained a mutation GGA➔GAA[125Gly➔Glu] in *mrpl-2*, the *C*. *elegans* homolog of MRPL2 mitochondrial ribosomal protein L2 subunit (Fig 1E). Consistently, knockdown of MRPL-2 and other mitochondrial ribosome subunits was previously found to activate the UPR^mt^ [3,5]. For the *osa5* allele we identified a GAT➔AAT[329Asp➔Asn] mutation in *pdr-1*, the *C*. *elegans* homolog of Parkin (Fig 1E). Reintroducing the wild-type *sucg-1* or *mrpl-2* gene locus by germline transformation could rescue UPR^mt^ activity back to wild-type levels for *osa2* and *osa3*, respectively (S2A and S2B Fig). However, re-introduction of the wild-type *pdr-1* locus into *osa5* animals did not rescue UPR^mt^ activity to wild-type levels (S2C Fig). Instead, germline transformation of wild-type animals with the *pdr-1* gene locus containing the *osa5* mutation was sufficient to induce the UPR^mt^ (S2C Fig). In contrast, germline transformation of wild-type animals with the wild-type *pdr-1* gene locus did not induce the UPR^mt^ (S2C Fig). Together, this suggests that *osa5* is a novel dominant allele of *pdr-1*. We were unable to map the gene responsible for the activation of the UPR^mt^ in *osa4* animals and therefore examined the connection between diet, genetic background, and UPR^mt^ activation using *mrpl-2(osa3)* animals.

### Diet influences mitochondrial function in *mrpl-2(osa3)* animals

**Fig 2:**
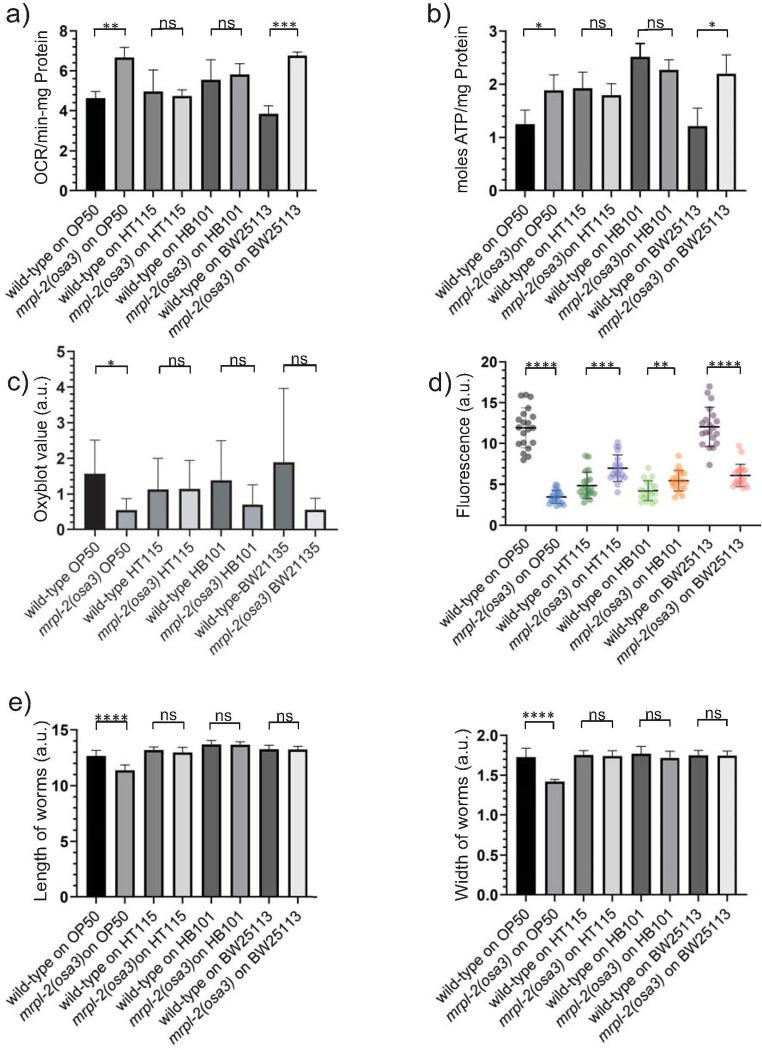
Mitochondrial function is altered in *mrpl-2(osa3)* animals in a diet-dependent manner. (A) Oxygen consumption rate determination for wild-type and *mrpl-2(osa3)* animals fed *E*. *coli* OP50, HT115, HB101, and BW25113. Oxygen consumption was normalized to total protein content (mean ±SD; *n* = 3); ns denotes not significant, ** denotes p ≤ 0.01, *** denotes p ≤ 0.001 (Student’s *t* test). (B) ATP production quantification for wild-type and *mrpl-2(osa3)* animals fed *E*. *coli* OP50, HT115, HB101, and BW25113. ATP levels are normalized to total protein content (mean ±SD; *n* = 3); ns denotes not significant, * denotes p ≤ 0.05 (Student’s *t* test). (C) Oxidative protein modification determination using the OxyBlot assay. OxyBlot values were normalized to actin for each sample and represented as arbitrary units (A.U.). (mean ± SD; n = 5); ns denotes not significant, * denotes p ≤ 0.05 (Student’s *t* test). (D) Mitochondrial membrane potential determination using TMRE and quantification for wild-type and *mrpl-2(osa3)* animals fed *E*. *coli* OP50, HT115, HB101, and BW25113 reflected as arbitrary units (A.U.). (mean ±SD; *n* ≥ 20 worms); ** denotes p ≤ 0.01, *** denotes p ≤ 0.001, **** denotes p ≤ 0.0001 (Student’s *t* test). (E) Animal size quantification of wild-type and *mrpl-2(osa3)* animals fed *E*. *coli* OP50, HT115, HB101, and BW25113. Animal size is expressed as the length and width of each animal and represented as arbitrary units (A.U.); (mean ±SD; *n* ≥ 20 worms); ns denotes not significant, **** denotes p ≤ 0.0001 (Student’s *t* test).

We next examined various parameters of mitochondrial function in wild-type and *mrpl-2(osa3)* animals fed the various *E*. *coli* diets. We first measured oxygen consumption levels which were surprisingly increased in *mrpl-2(osa3)* animals fed *E*. *coli* OP50 and BW25113, but not HT115, and HB101 (Fig 2A). We observed a similar trend when measuring ATP levels which were increased in *mrpl-2(osa3)* animals fed *E*. *coli* OP50 and BW25113, whereas no change occurred with HT115 and HB101 (Fig 2B). Increased mitochondrial activity or dysfunction can generate toxic reactive oxygen species (ROS) that perturbs protein homeostasis through the formation of carbonyl modifications. We therefore used the OxyBlot system which assesses protein carbonylation as a measure of oxidative damage. Interestingly, *mrpl-2(osa3)* animals fed a diet of *E*. *coli* OP50 showed reduced oxidative damage compared to wild-type (Figs 2C and S3). No change in oxidative damage was observed when *mrpl-2(osa3)* animals were fed diets of *E*. *coli* HT115, HB101, or BW25113 (Figs 2C and S3). Lastly, mitochondrial function was also examined through an assessment of mitochondrial membrane potential. Consistent with an activation of the UPR^mt^, *mrpl-2(osa3)* animals displayed reduced mitochondrial membrane potential when fed a diet of *E*. *coli* OP50 (Figs 2D and S4A). Mild increases in mitochondrial membrane potential were observed when *mrpl-2(osa3)* animals were fed diets of *E*. *coli* HT115 and HB101 (Figs 2D and S4A). Surprisingly, mitochondrial membrane potential was also reduced when *mrpl-2(osa3)* animals were fed a diet of *E*. *coli* BW25113 despite no observable activation of the UPR^mt^ (Figs 2D and S4A).

Slowed developmental rates are often a consequence of mitochondrial stress. However, we did not observe any significant change in developmental rates between wild-type and *mrpl-2(osa3)* fed on the various diets (S4B Fig). While the development of *mrpl-2(osa3)* animals was not significantly different, we did notice that *mrpl-2(osa3)* animals appeared thinner and overall slightly smaller when fed *E*. *coli* OP50 whereas no difference was observed when these animals were fed *E*. *coli* HT115, HB101, or BW25113 (Fig 2E).

### *mrpl-2(osa3)* extends lifespan and increases host resistance in a diet-dependent manner

**Fig 3:**
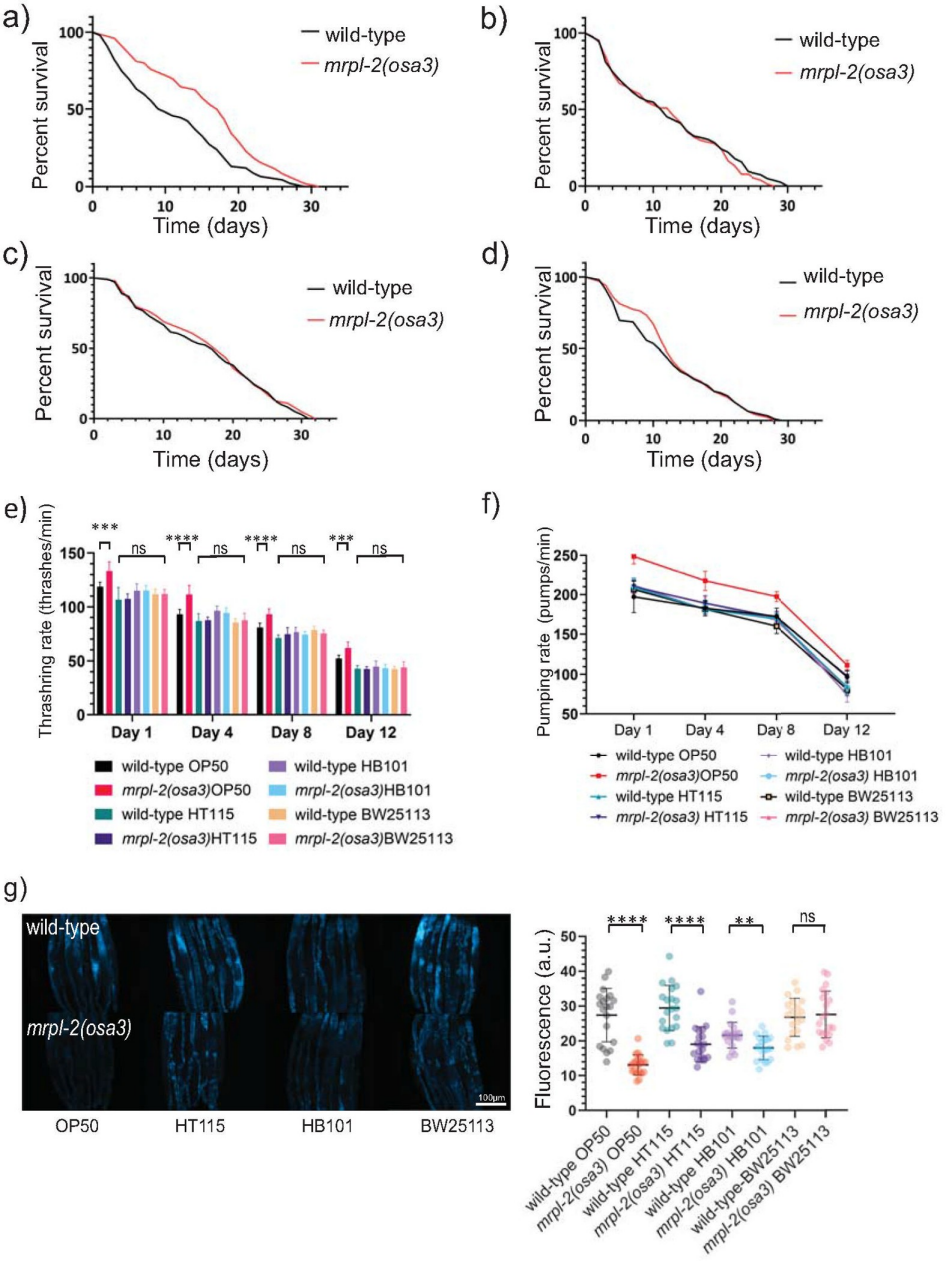
Diet-type determines lifespan in *mrpl-2(osa3)* animals. (A-D) Lifespans of wild-type and *mrpl-2(osa3)* animals fed *E*. *coli* (A) OP50, (B) HT115, (C) HB101, and (D) BW25113. See S1 Table for all lifespan assay statistics. (E) Whole body thrashing rate quantification of wild-type and *mrpl-2(osa3)* animals fed *E*. *coli* OP50, HT115, HB101, and BW25113. (mean ±SD; *n* ≥ 10 worms); ns denotes not significant, *** denotes p ≤ 0.001, **** denotes p ≤ 0.0001 (Student’s *t* test). (F) Pharyngeal pumping rate quantification of wild-type and *mrpl-2(osa3)* animals fed *E*. *coli* OP50, HT115, HB101, and BW25113 (mean ±SD; *n* ≥ 10 worms). (G) Photomicrographs and quantification of gut autofluorescence in wild-type and *mrpl-2(osa3)* animals. Quantification of fluorescence intensities expressed as arbitrary units (A.U.); ns denotes not significant, ** denotes p ≤ 0.01, **** denotes p ≤ 0.0001 (Student’s *t* test).

We next examined lifespans of wild-type and *mrpl-2(osa3)* animals fed the various diets. As expected with the activation of the UPR^mt^, *mrpl-2(osa3)* animals lived longer than wild-type when fed *E*. *coli* OP50 (Fig 3A). No differences in lifespan between wild-type and *mrpl-2(osa3)* were observed when fed *E*. *coli* HT115, HB101, and BW25113 which is consistent with a lack of UPR^mt^ activation on these diets (Fig 3B–3D). Importantly, an extrachromosomal array consisting of the wild-type *mrpl-2* gene locus suppressed the increased longevity of *mrpl-2(osa3)* animals in two independent transgenic lines resulting in normal (wild-type) lifespan levels (S5 Fig), indicating that mutation in *mrpl-2* is the cause of the observed lifespan extension.

We then measured various physiological markers of aging between wild-type and *mrpl-2(osa3)* under the different diets. We first measured thrashing rates which reflects body wall muscle integrity. We find that muscle function decline was less in *mrpl-2(osa3)* animals compared to wild-type when fed *E*. *coli* OP50 whereas no differences in thrashing rate was observed when *mrpl-2(osa3)* animals were fed other diets (Fig 3E). We then quantified pharyngeal pumping which is a reflection of the rate of food intake and found it to be higher in aged *mrpl-2(osa3)* animals compared to wild-type when fed *E*. *coli* OP50 whereas no difference was detected when fed *E*. *coli* HT115, HB101, and BW25113 (Fig 3F). Lastly, accumulation of lipofuscin is a hallmark of aging in *C*. *elegans* that is reflected as intestinal autofluorescence [19]. The greatest reduction in autofluorescence was observed when *mrpl-2(osa3)* animals were fed *E*. *coli* OP50 but only a mild difference was observed when fed *E*. *coli* HT115, HB101, and no difference for BW25113 (Fig 3G). Therefore, *mrpl-2(osa3)* slows physiological markers of aging when fed a diet of *E*. *coli* OP50.

**Fig 4:**
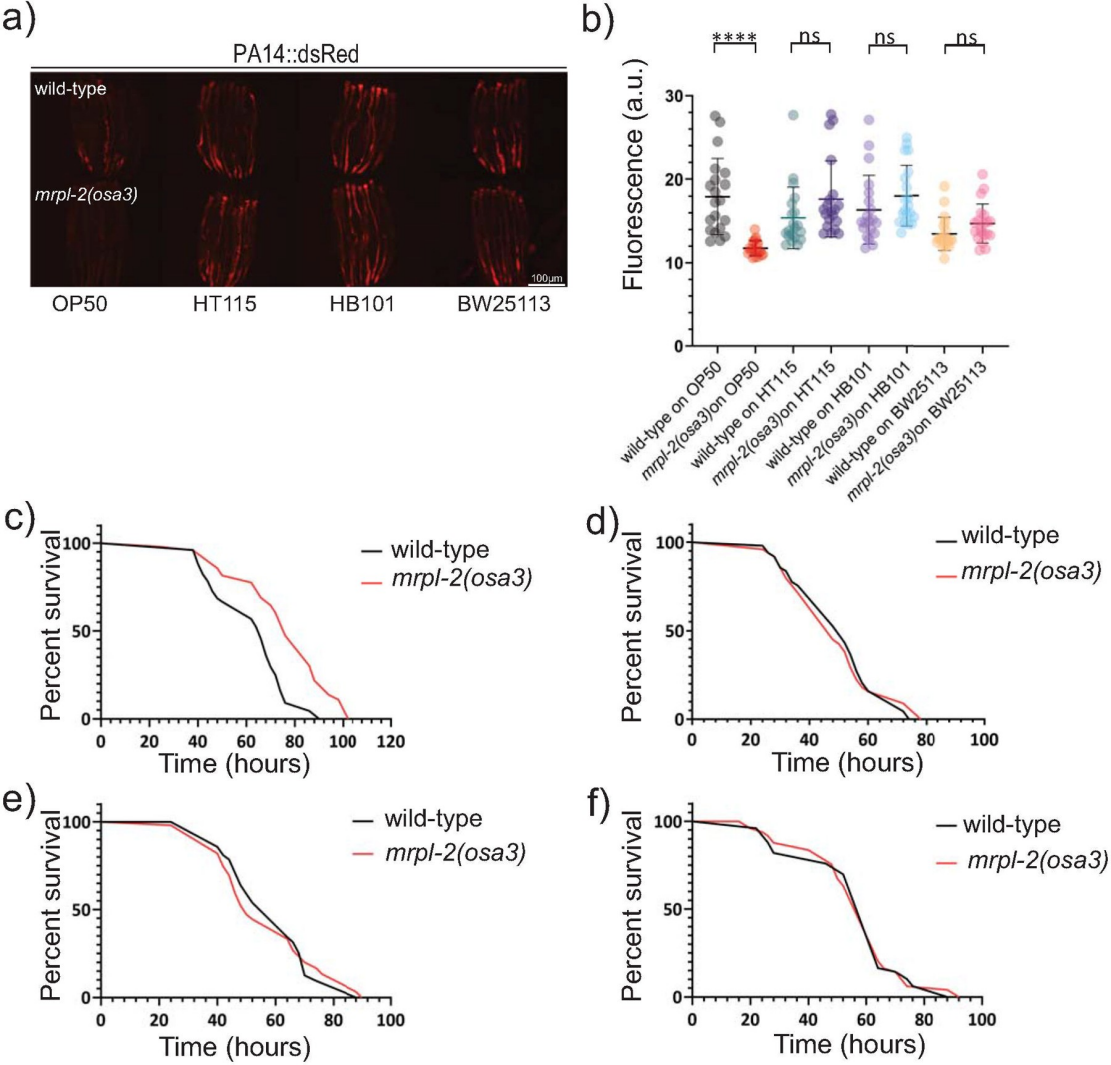
*mrpl-2(osa3)* animals survive longer during infection depending on their prior diet. (A, B) Photomicrographs and quantification of *P*. *aeruginosa* PA14-dsRed expression of infected wild-type or *mrpl-2(osa3)* animals previously fed different diets of *E*. *coli*. Quantification of fluorescence intensities expressed as arbitrary units (A.U.); (mean ±SD; *n* ≥ 20 worms); ns denotes not significant, **** denotes p ≤ 0.0001 (Student’s *t* test). (C-F) Survival analysis of wild-type and *mrpl-2(osa3)* animals infected with *P*. *aeruginosa*. *E*. *coli* diets prior to *P*. *aeruginosa* infection are (C) OP50, (D) HT115, (E) HB101, and (F) BW25113. See S1 Table for all survival assay statistics.

Next, we examined the effect of diet on the ability of *mrpl-2(osa3)* animals to survive infection with the opportunistic pathogen *Pseudomonas aeruginosa* [13]. Consistent with increased host resistance, pathogen colonization was lower in *mrpl-2(osa3)* animals that were previously fed *E*. *coli* OP50 whereas similar pathogen colonization levels were observed in *mrpl-2(osa3)* animals previously fed *E*. *coli* HT115, HB101, and BW25113 (Fig 4A and 4B). Accordingly, *mrpl-2(osa3)* animals survived significantly longer than wild-type animals during infection with *P*. *aeruginosa* if they were previously fed a diet of *E*. *coli* OP50 (Fig 4C). No difference in host survival was observed between wild-type and *mrpl-2(osa3)* animals when each were previously fed diets of *E*. *coli* HT115, HB101, or BW25113 (Fig 4D–4F).

We next wished to confirm that mild dysfunction to mitochondrial translation was synergizing specifically with a diet of *E*. *coli* OP50 to induce the UPR^mt^. Here, we treated wild-type animals with doxycycline which was previously found to inhibit mitochondrial translation, activate the UPR^mt^ and increase *C*. *elegans* lifespan [3]. We find that exposure of wild-type animals to a mild dose of doxycycline activated the UPR^mt^ when fed a diet of *E*. *coli* OP50 but not HT115, HB101, or BW25113 (S6A Fig). In addition, exposure to a mild dose of doxycycline increased the lifespan of wild-type animals only when they were fed a diet of *E*. *coli* OP50, whereas no difference was observed when fed *E*. *coli* HT115, HB101, or BW25113 (S6B–S6E Fig). Thus, mild disruption to mitochondrial translation either through genetic (*mrpl-2(osa3)*) or chemical (doxycycline) means activates the UPR^mt^ and extends lifespan in a diet-dependent manner.

Together, our data suggests an interplay exists between diet and the *mrpl-2(osa3)* genetic background that drives activation of the UPR^mt^. Hereafter, we focus on the diets of *E*. *coli* OP50 and HT115 to dissect the mechanisms behind this interaction.

### Vitamin B12 availability synergizes with *mrpl-2(osa3)* to activate the UPR^mt^

**Fig 5:**
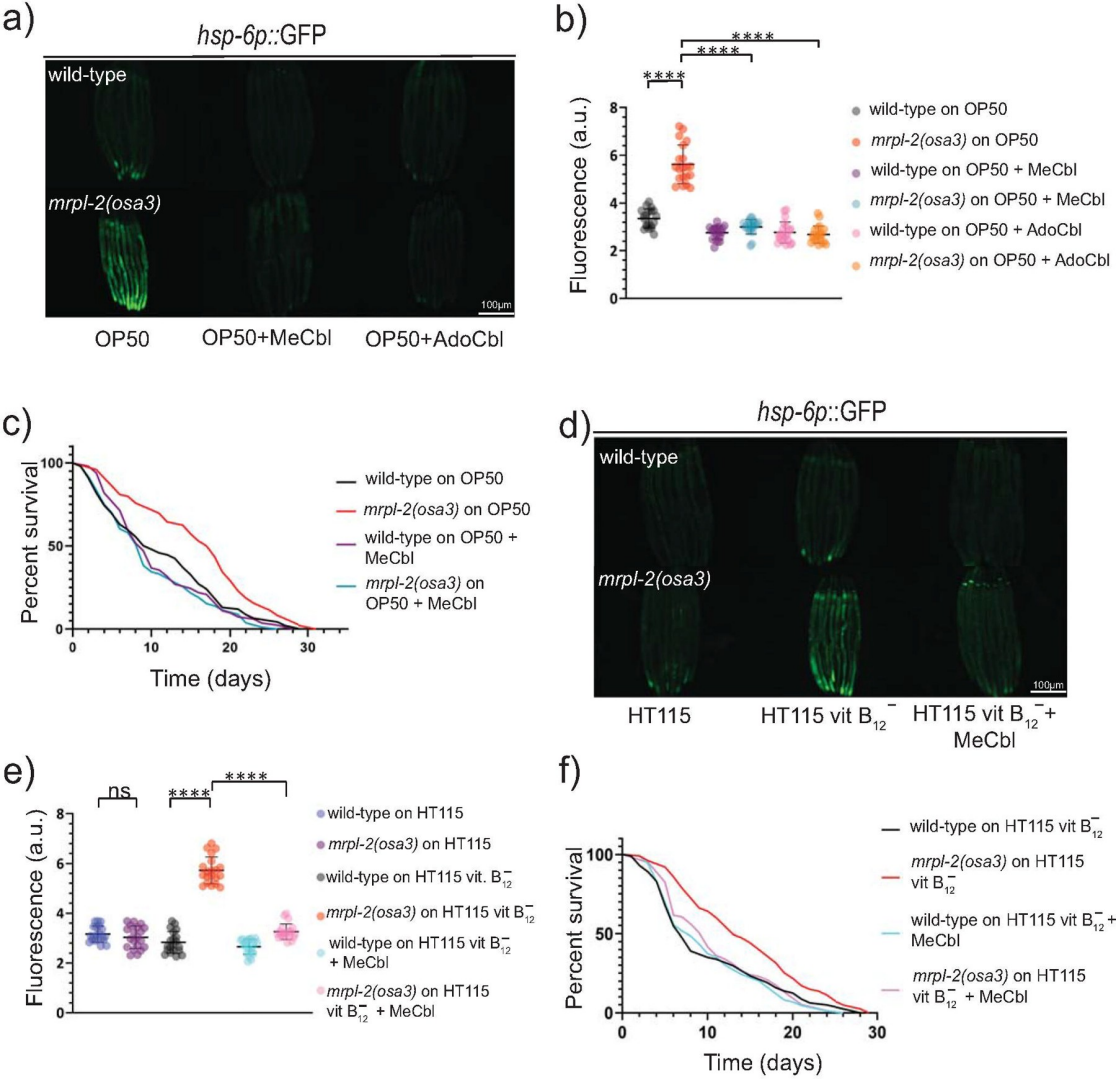
Vitamin B12 levels determine the activation of the UPR^mt^ in *mrpl-2(osa3)* animals under different diets. (A, B) Photomicrographs and quantification of *hsp-6p*::GFP expression in wild-type and *mrpl-2(osa3)* animals fed *E*. *coli* OP50 in the presence or absence of 0.2 μg/ml methylcobalamin or adenosylcobalamin. Quantification of fluorescence intensities expressed as arbitrary units (A.U.); (mean ±SD; *n* ≥ 20 worms); **** denotes p ≤ 0.0001 (Student’s *t* test). (C) Lifespans of wild-type and *mrpl-2(osa3)* animals fed *E*. *coli* OP50 in the presence or absence of 0.2 μg/ml methylcobalamin. (D, E) Photomicrographs and quantification of *hsp-6p*::GFP expression in wild-type and *mrpl-2(osa3)* animals fed vitamin B12-restricted *E*. *coli* HT115 in the presence or absence of 0.2 μg/ml methylcobalamin. Quantification of fluorescence intensities expressed as arbitrary units (A.U.); (mean ±SD; *n* ≥ 20); ns denotes not significant, **** denotes p ≤ 0.0001 (Student’s *t* test). (F) Lifespans of wild-type and *mrpl-2(osa3)* animals fed vitamin B12-restricted *E*. *coli* HT115 in the presence or absence of 0.2 μg/ml methylcobalamin.

We next explored the mechanism behind the relationship of diet and the activation of the UPR^mt^ in *mrpl-2(osa3)* animals. A recent study found that *E*. *coli* OP50 is deficient in the nutrient vitamin B12 compared to *E*. *coli* HT115 [20]. We hypothesized that lower levels of vitamin B12 in the *E*. *coli* OP50 diet might interact with *mrpl-2(osa3)* to drive the activation of the UPR^mt^. Indeed, wild-type animals fed a diet of *E*. *coli* OP50 had lower levels of vitamin B12 compared to those fed a diet of *E*. *coli* HT115 (S7 Fig). We therefore supplemented the *E*. *coli* OP50 diet with two biologically active forms of vitamin B12, methylcobalamin and adenosylcobalamin, which are used as cofactors in the activation of associated effectors. Interestingly, we found that an *E*. *coli* OP50 diet supplemented with methylcobalamin or adenosylcobalamin was able to suppress the activation of *hsp-6p*::GFP in *mrpl-2(osa3)* animals (Fig 5A and 5B). The finding that either methylcobalamin or adenosylcobalamin could suppress the activation of the UPR^mt^ is likely due to their ability to be interconverted [21]. We next measured lifespans of wild-type or *mrpl-2(osa3)* animals fed an *E*. *coli* OP50 diet supplemented with methylcobalamin. Consistent with attenuating the activation of the UPR^mt^, methylcobalamin supplementation suppressed the extended lifespan of *mrpl-2(osa3)* fed a diet of *E*. *coli* OP50 (Fig 5C).

Next, we examined the consequences of restricting vitamin B12 levels from the diet of *E*. *coli* HT115 using a previously established technique [22]. Restricting vitamin B12 levels was able to activate the UPR^mt^ in *mrpl-2(osa3)* animals but not in the wild-type, similar to when they were fed *E*. *coli* OP50 (Fig 5D and 5E). Supplementing with methylcobalamin attenuated UPR^mt^ activity in vitamin B12-restricted *mrpl-2(osa3)* animals (Fig 5D and 5E). Consistent with an activation of the UPR^mt^, reducing vitamin B12 levels from the HT115 diet also extended the lifespan of *mrpl-2(osa3)* animals, which could be suppressed with methylcobalamin supplementation (Fig 5F). Together, our data suggest that the effect of diet on the activation of the UPR^mt^ in the *mrpl-2(osa3)* background is due to differences in vitamin B12 content.

### Mitonuclear imbalance and methionine restriction act in a common pathway to promote lifespan extension

**Fig 6:**
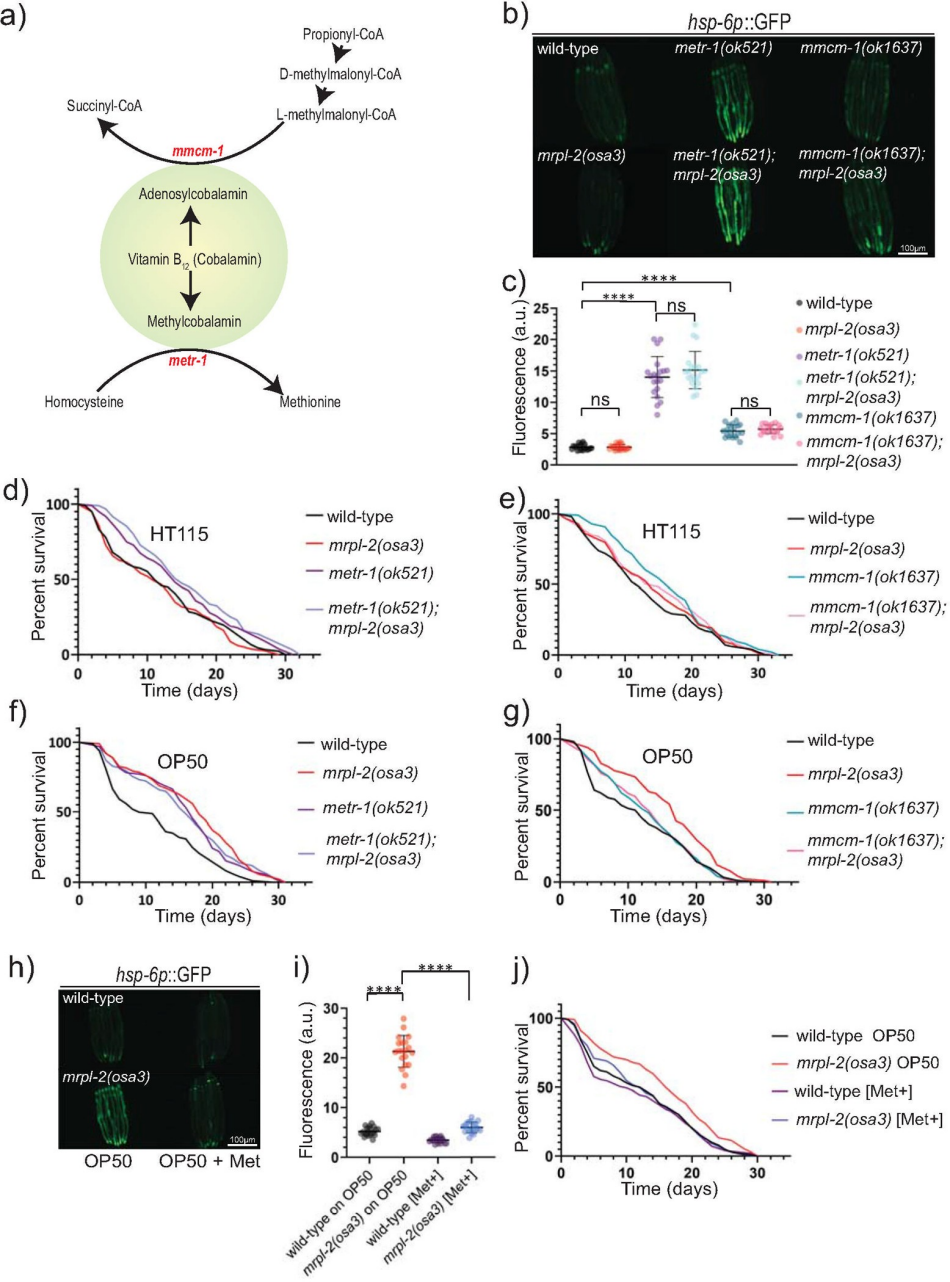
Methionine supplementation suppresses UPR^mt^ activation in *mrpl-2(osa3)* animals fed a vitamin B12 deficient diet. (A) Schematic illustration of vitamin B12-dependent metabolic pathways. (B, C) Photomicrographs and quantification of *hsp-6p*::GFP expression of wild-type, *mrpl-2(osa3)*, *metr-1(ok521)*, *mmcm-1(ok1637)*, *metr-1(ok521); mrpl-2(osa3)*, and *mmcm-1(ok1637); mrpl-2(osa3)* fed an *E*. *coli* HT115 diet. Quantification of fluorescence intensities expressed as arbitrary units (A.U.); (mean ±SD; *n* ≥ 20); ns denotes not significant, **** denotes p ≤ 0.0001 (Student’s *t* test). (D) Lifespans of wild-type, *mrpl-2(osa3)*, *metr-1(ok521)*, *metr-1(ok521); mrpl-2(osa3)* fed an *E*. *coli* HT115 diet. (E) Lifespans of wild-type, *mrpl-2(osa3)*, *mmcm-1(ok1637)*, *mmcm-1(ok1637); mrpl-2(osa3)* fed an *E*. *coli* HT115 diet. (F) Lifespans of wild-type, *mrpl-2(osa3)*, *metr-1(ok521)*, *metr-1(ok521); mrpl-2(osa3)* fed an *E*. *coli* OP50 diet. (G) Lifespans of wild-type, *mrpl-2(osa3)*, *mmcm-1(ok1637)*, *mmcm-1(ok1637); mrpl-2(osa3)* fed an *E*. *coli* OP50 diet. (H, I) Photomicrographs and quantification of *hsp-6p*::GFP expression of wild-type and *mrpl-2(osa3)* fed an *E*. *coli* OP50 diet in the presence or absence of 10 mM methionine. Quantification of fluorescence intensities expressed as arbitrary units (A.U.); (mean ±SD; *n* 20); denotes p 0.0001 (Student’s *t* test). (J) Lifespans of wild-type and *mrpl-2(osa3)* fed an *E*. *coli* OP50 diet in the presence or absence of 10 mM methionine.

Vitamin B12 is used as a cofactor for two separate enzymes: methionine synthase which mediates the conversion of homocysteine to methionine during the S-adenosylmethionine/methionine cycle, and methylmalonyl-CoA mutase which converts L-methylmalonyl-CoA to succinyl-CoA (Fig 6A) [21]. Therefore, we tested the effects of genetically disabling these pathways under a vitamin B12 replete diet of HT115 using mutants in methionine synthase, (*metr-1(ok521)*) and methylmalonyl-CoA mutase (*mmcm-1(ok1637)*). Interestingly, *metr-1(ok521)* or *mmcm-1(ok1637)* individual loss of function mutants activated the UPR^mt^ when they were fed a diet of HT115 (Fig 6B and 6C). However, no further enhancement of the UPR^mt^ was observed for each mutation in combination with the *mrpl-2(osa3)* background (Fig 6B and 6C).

We then examined the effects of *mmcm-1(ok1637)* or *metr-1(ok521)* on the lifespan of *mrpl-2(osa3)* fed a vitamin B12-replete diet of *E*. *coli* HT115 or when fed a diet of *E*. *coli* OP50. Neither *mmcm-1(ok1637)* or *mmcm-1(ok1637); mrpl-2(osa3)* animals exhibited any significant change in lifespan fed a diet of *E*. *coli* HT115 (Fig 6E). Unexpectedly, a modest but significant increase in lifespan was observed in *metr-1(ok521)* single mutants fed *E*. *coli* HT115 (Fig 6D), previously found to have normal rates of aging when fed *E*. *coli* OP50 [23]. However, there was no significant difference in lifespan between *metr-1(ok521)* and *metr-1(ok521); mrpl-2(osa3)* mutants fed a diet of *E*. *coli* HT115 (Fig 6D). In contrast, a greater lifespan extension was observed in *metr-1(ok521)* animals fed the *E*. *coli* OP50 diet to levels comparable with *E*. *coli* OP50-fed *mrpl-2(osa3)* (Fig 6F). However, and interestingly, no further extension was observed in *metr-1(ok521); mrpl-2(osa3)* double mutant animals fed *E*. *coli* OP50 (Fig 6F). Surprisingly, although the lifespan of *mmcm-1(ok1637)* was not significantly extended compared to wild-type when fed a diet of *E*. *coli* OP50, *mmcm-1(ok1637)* nonetheless reduced the lifespan extension of *mrpl-2(osa3)* (Fig 6G). This suggests that *mrpl-2(osa3)* extends lifespan when fed a diet of OP50 in a MMCM-1-dependent manner.

Because the lifespan extensions of *mrpl-2(osa3)* and *metr-1(ok521)* animals were not additive in the double mutant background suggests that mitonuclear imbalance and methionine restriction may use a common pathway(s) to regulate aging. Indeed, wild-type animals fed a diet of *E*. *coli* OP50 were found to have lower levels of methionine compared to those fed a diet of *E*. *coli* HT115 (S8 Fig). In addition, supplementation with methionine, but not other amino acids, resulted in a near complete suppression of the UPR^mt^ in *mrpl-2(osa3)* animals fed a diet of *E*. *coli* OP50 (Figs 6H and 6I and S9). Consistently, methionine supplementation also completely suppressed the increase in longevity observed with *mrpl-2(osa3)* animals fed *E*. *coli* OP50 (Fig 6J).

### ATFS-1 mediates extended lifespan resulting from mitonuclear imbalance or methionine restriction

**Fig 7:**
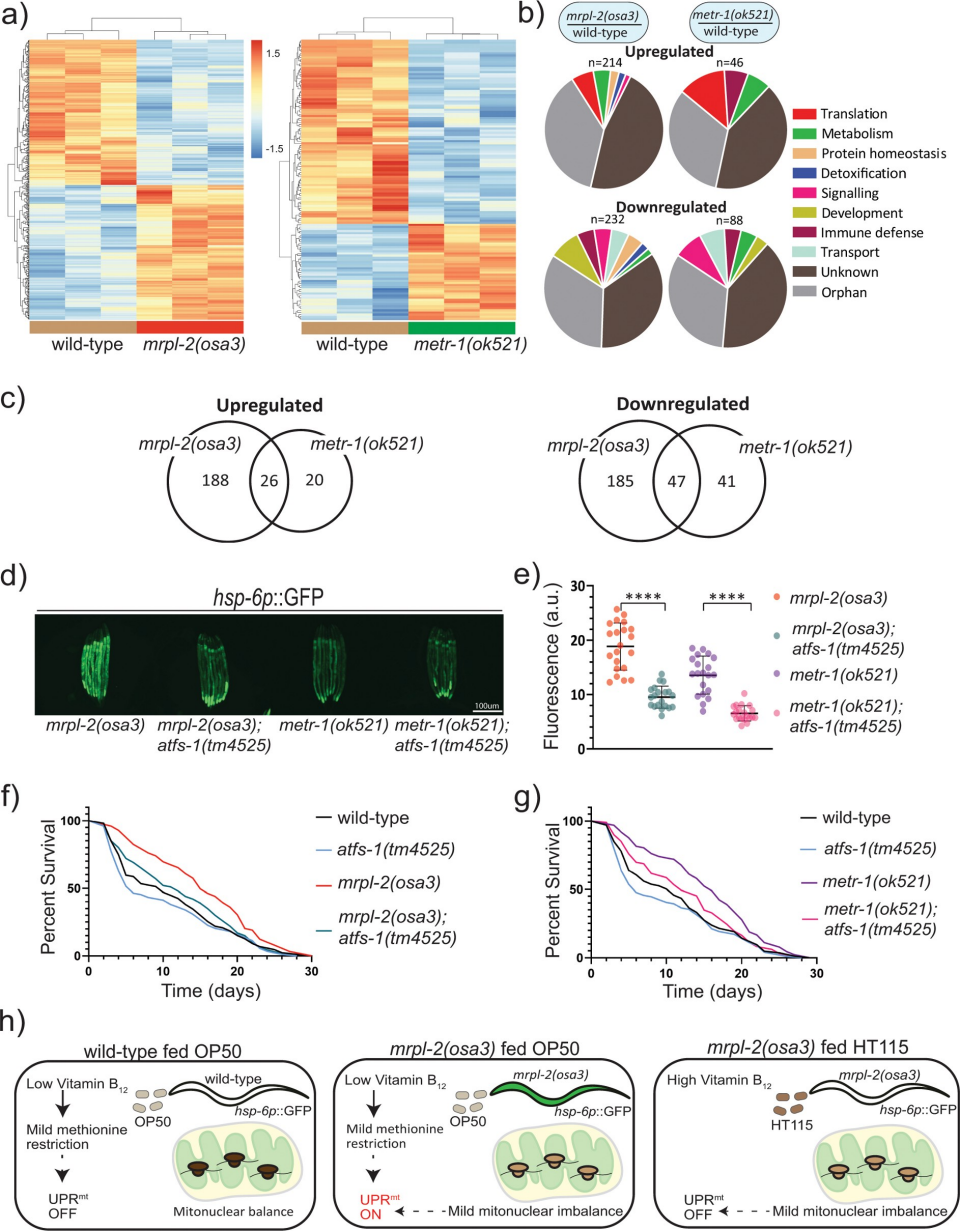
ATFS-1/UPR^mt^ mediate the lifespan extension of mitonuclear imbalance and methionine restriction. (A) Heat maps representing gene expression changes in wild-type, *mrpl-2(osa3)*, and *metr-1(ok521)* fed an *E*. *coli* OP50 diet. Genes were considered as differentially expressed if there was a significance difference of p ≤ 0.05 (after Benjamini-Hochberg correction). (B) Functional categories of differentially expressed genes. (C) Venn diagram of differentially expressed genes shared between *mrpl-2(osa3)* and *metr-1(ok521)*. (D, E) Photomicrographs and quantification of *hsp-6p*::GFP expression for *mrpl-2(osa3)*, *mrpl-2(osa3)*; *atfs-1(tm4525)*, *metr-1(ok521)*, *and metr-1(ok521); atfs-1(tm4525)* fed a diet of *E*. *coli* OP50. Quantification of fluorescence intensities expressed as arbitrary units (A.U.); (mean ±SD; *n* ≥ 20); **** denotes p ≤ 0.0001 (Student’s *t* test). (F) Lifespans of wild-type, *mrpl-2(osa3)*, *atfs-1(tm4525)*, and *mrpl-2(osa3)*; *atfs-1(tm4525)* fed a diet of *E*. *coli* OP50. (G) Lifespans of wild-type, *metr-1(ok521)*, *atfs-1(tm4525)*, and *metr-1(ok521)*; *atfs-1(tm4525)* fed a diet of *E*. *coli* OP50. (H) Model. Wild-type animals fed a diet of *E*. *coli* OP50 experience a mild vitamin B12 restriction which reduces methionine synthase activity resulting in a subtle methionine restriction that is insufficient to activate the UPR^mt^. However, the UPR^mt^ is activated in combination with the mild mitonuclear imbalance of the *mrpl-2(osa3)* reduction of function mutant which results in extended lifespan. In contrast, methionine supply is higher when fed a vitamin B12 replete diet of *E*. *coli* HT115, and therefore the mild mitonuclear imbalance of *mrpl-2(osa3)* mutant alone is incapable of inducing the UPR^mt^ in this scenario resulting in animals that display normal aging rates.

We next performed transcriptomics to evaluate the changes in gene expression occurring during mitonuclear imbalance and methionine restriction using *mrpl-2(osa3)* and *metr-1(ok521)* animals, respectively. We hypothesized that *mrpl-2(osa3)* and *metr-1(ok521)* extended lifespan using at least one common pathway since the lifespan of the double mutant was not additive. Using a cutoff *p*-value of <0.05 (after Benjamini-Hochberg correction), our transcriptomic analysis indicated that there were relatively fewer genes that were differentially expressed in *metr-1(ok521)* animals relative to wild-type animals compared to those differentially expressed in *mrpl-2(osa3)* (Fig 7A–7C and S2 Table). However, there was considerable overlap between the genes that were differentially expressed in each genetic background. Of the 46 genes that were upregulated in *metr-1(ok521)*, 26 were shared with *mrpl-2(osa3)* (Fig 7C). Similarly, of the 88 genes that were downregulated in *metr-1(ok521)* animals, 47 were in common with *mrpl-2(osa3)* (Fig 7C).

Since both mitonuclear imbalance and methionine restriction were able to induce the UPR^mt^, we suspected that this pathway may be required for their effects on lifespan. Therefore, we next examined whether the increased longevity of *mrpl-2(osa3)* or *metr-1(ok521)* animals required the UPR^mt^ by using the *atfs-1(tm4525)* reduction of function mutant. As expected, loss of ATFS-1 reduced the activation of the UPR^mt^ in OP50-fed *mrpl-2(osa3)* and *metr-1(ok521)* animals (Fig 7D and 7E). Also, loss of ATFS-1 suppressed the increase in animal lifespan observed with *mrpl-2(osa3)* fed a diet of *E*. *coli* OP50 while having no significant effect in an otherwise wild-type background (Fig 7F). This is consistent with previous reports illustrating the need for the UPR^mt^ for mitonuclear imbalance-induced lifespan extension [3]. Interestingly, *atfs-1* reduction of function also suppressed the increase in lifespan resulting from *metr-1(ok521)* methionine restriction (Fig 7G). Thus, ATFS-1 and the UPR^mt^ are required for lifespan extension resulting from both mitonuclear imbalance and methionine restriction.

## Discussion

Our data suggests that the *mrpl-2(osa3)* allele is a reduction of function allele that creates a sensitized background for UPR^mt^ activation and that the type of diet can tip the balance in its favor (Fig 7H). Vitamin B12 availability appears to be the metabolite that determines whether a diet will activate the UPR^mt^ in the *mrpl-2(osa3)* background via the methionine synthase pathway. Consistently, under vitamin B12 replete conditions, the level of methionine restriction is insufficient to activate the UPR^mt^ in the *mrpl-2(osa3)* sensitized background and to extend lifespan. Interestingly, we find that disruption to the vitamin B12-dependent methionine synthesis pathway via inactivation of METR-1 also activates the UPR^mt^ and can extend animal lifespan. While bacterial diet is the main source of methionine for *C*. *elegans*, this amino acid can also be synthesized to a small degree via METR-1 and thus, reduced function of METR-1 results in a sensitized condition of methionine restriction [23]. Importantly, we find that a reduction of function mutation in *mrpl-2* and mild methionine restriction use a common mechanism of longevity regulation, including the need of the UPR^mt^ regulator ATFS-1.

Diet is an important determinant controlling organismal aging [24]. While dietary restriction has long been appreciated in mediating lifespan extension [25,26], there is a growing recognition that the *type* of diet and genetic background of the host can interact in order to control aging rates. This has been observed in a number of cases in *C*. *elegans*. For example, a mutation in the *C*. *elegans* 1-pyrroline-5-carboxylate (P5C) dehydrogenase homolog *alh-6*, which mediates the conversion of P5C to glutamate during proline metabolism in mitochondria, displays reduced lifespan when fed on the *E*. *coli* strain OP50, but not when fed with the strain HT115 [27]. The reduced lifespan observed for *alh-6* mutants grown on *E*. *coli* OP50 is due to increased accumulation of P5C that results in mitochondrial dysfunction. A second example of how gene-diet can affect lifespan is seen with a mutation in the *rict-1* gene, the *C*. *elegans* homolog of a component of the Target of Rapamycin complex 2 (TORC2). Lifespan of *rict-1* mutants fed *E*. *coli* OP50 is reduced compared to that observed using the *E*. *coli* strain HB101 [28]. Interestingly, *rict-1* mutants tend to avoid the HB101 strain more often than OP50 resulting in less feeding and a dietary restriction-induced increase in longevity. More recently, loss of the kinase FLR-4 was shown to increase *C*. *elegans* longevity when fed *E*. *coli* HT115 but not OP50, through diet-specific activation of p38 MAPK and xenobiotic gene expression [29]. Our study indicates that a specific genetic background can synergize with a diet of low vitamin B12 to promote lifespan extension via activation of the UPR^mt^. It is interesting that vitamin B12 restriction was previously shown to reduce, rather than increase, lifespan duration [22]. It is possible that the sensitized *mrpl-2(osa3)* background requires a lesser degree of vitamin B12 restriction that promotes, rather than antagonizes, lifespan. Indeed, the activation of the UPR^mt^ and increase in longevity of *mrpl-2(osa3)* animals required less vitamin B12 restriction (four generations of growth on vitamin B12-deficient *E*. *coli*) than was previously reported which reduced the lifespan of wild-type animals (following five generations of growth on vitamin B12-deficient *E*. *coli*) [22]. Also interesting is that a vitamin B12-replete diet of HT115 was previously shown to promote host protection against *P*. *aeruginosa* infection compared to a vitamin B12-restricted diet of OP50 [20], whereas our study discovered the opposite trend. However, the aforementioned protection occurred in the context of *P*. *aeruginosa*-mediated liquid-killing which reduces animal survival through the production of iron-binding siderophores [30]. In our current study, reduced vitamin B12 supply allowed *mrpl-2(osa3)* animals to survive longer during *P*. *aeruginosa* slow-killing which results from pathogen colonization of the gut [31]. This suggests that the beneficial effects of gene-diet interactions are context-dependent.

We also show that a reduction of function mutation in a mitochondrial ribosome gene converges with methionine restriction to promote lifespan at the level of the UPR^mt^. Mitonuclear imbalance was previously shown to increase animal longevity which required the UPR^mt^ regulator UBL-5 [3]. Our study supports this finding by demonstrating a requirement of ATFS-1 for the extended lifespan of *mrpl-2(osa3)* fed a low vitamin B12 diet. Also, while an association between methionine restriction and lifespan extension has previously been reported [32], to our knowledge this is the first connection between this pathway and the activation of the UPR^mt^. While the mechanism behind the activation of the UPR^mt^ resulting from methionine restriction was not explored, we do show a requirement for ATFS-1 in mediating the observed extension in longevity. The association between ATFS-1/UPR^mt^ and the extended lifespan that is observed with mitochondrial stress remains controversial [5,12,18,33]. Certain conditions that activate the UPR^mt^ are associated with extended lifespan and require ATFS-1 or other regulators of this stress response pathway. This includes mitonuclear imbalance [3], ETC dysfunction [12], and more recently from the loss of two neuronal epigenetic regulators [34]. Contrary to these findings, ATFS-1 had no role in regulating the lifespan increase observed with RNAi knockdown of the cytochrome c oxidase 1 gene *cco-1*, despite it reducing the expression of the UPR^mt^ reporter [5]. Also, ATFS-1 was not required for the lifespan extension observed with transaldolase inhibition despite an activated UPR^mt^ [33]. Furthermore, constitutive activation of ATFS-1 does not extend animal lifespan but rather, accelerates aging [5]. Our finding that ATFS-1 is required for the increased longevity of *mrpl-2(osa3)* animals is in line with previous observations demonstrating a requirement of the UPR^mt^ for mitonuclear imbalance-induced longevity [3]. We have also shown that mild methionine restriction could also activate the UPR^mt^ and extend lifespan in an ATFS-1-dependent manner. One possibility is that the requirement of ATFS-1 and the UPR^mt^ for longevity is highly context-dependent and thus is only revealed during specific types of mitochondrial stress such as the conditions presented in this study. Furthermore, the regulation of lifespan by the UPR^mt^ may also be dependent on the strength of mitochondrial stress encountered. For example, the matrix peptide exporter HAF-1 regulates the UPR^mt^ under conditions of mild mitochondrial stress but not under elevated levels of dysfunction [9,35]. Future work will now focus on identifying the mechanism of UPR^mt^ activation and lifespan extension occurring with methionine restriction which may help resolve these inconsistencies further.

## Materials and methods

### *C*. *elegans* and bacterial strains

*C*. *elegans* strains were maintained on nematode growth medium (NGM) using previously established methods [36] and cultured at 20°C. Various previously reported *C*. *elegans* strains were obtained from the Caenorhabditis Genetics Center (CGC) and include: N2 Bristol [36], *zcIs13*[*hsp-6p*::GFP], *metr-1(ok521)*, *mmcm-1(ok1637)*. Strains identified in this study include: *sucg-1(osa2)*, *mrpl-2(osa3)*, *osa4*, and *pdr-1(osa5)*. All mutant strains were backcrossed at least four times prior to use. Transgenic rescue worm strains were as follows: *sucg-1(osa2)* [*sucg-1*+], *mrpl-2(osa3)* [*mrpl-2*+], *pdr-1(osa5)* [*pdr-1*+], N2 [*pdr-1*+], and N2 [*pdr-1*(*osa5*)].

The following bacterial strains were also used in this study: *E*. *coli* strains OP50, HB101, HT115, and BW25113, as well as *P*. *aeruginosa* PA14 and *P*. *aeruginosa* PA14-dsRed. *E*. *coli* strains were obtained from the CGC and *P*. *aeruginosa* strains were a gift from Dr. Joao Xavier (MSKCC).

### EMS mutagenesis

Approximately 2000 SJ4100 animals were harvested from NGM plates with S-basal, washed twice to remove bacteria and resuspended in 2 ml S-basal. 2 ml of 2X ethyl methyl sulfonate (EMS) solution (60 μM) was added to worm suspension and placed on a rocker for 4 hrs. After mutagenesis, worms were washed three times with S-basal, resuspended in 0.5 ml S-basal and plated onto seeded NGM plates. After overnight incubation, 50 adult worms were singled out into NGM plates and allowed to grow until F2 generation (7 days). F2 worms showing green fluorescence were selected for further study.

### Germline transformation

We used germline transformation to rescue the phenotypes associated with *sucg-1(osa2)*, *mrpl-2(osa3)*, and *pdr-1(osa5)* using standard techniques [37]. PCR fragments were generated consisting of the promoter, open reading frame, and 3’UTR of each gene and microinjected at 10 ng/μl along with *Pmyo-2*::mCherry plasmid at 5 ng/μl as a co-injection marker. Promoter lengths used for germline transformation experiments were as follows: *mrpl-2* (533 bp), *sucg-1* (2153 bp), and *pdr-1* (1613 bp). Primer sequences used to PCR amplify each rescue fragment were as follows: *mrpl-2*: ttcacagccagactccaatg and gctatttgccgatttgtcgt, *sucg-1*: gcagctccttctgatcttgg and ggaagggtatgccattttga, *pdr-1*: gcgcctcttcatgattagca and catttgttgctgctgttgct. For [*pdr-1(osa5)*] rescue PCR, *pdr-1(osa5)* genomic DNA was used as a template. At least two independent transgenic lines were used from each transformation to test for rescue.

### Microscopy

All fluorescent reporter expression assays were conducted using a Zeiss Observer Z1 upright microscope. Worms were anesthetized using 2.5 mM sodium azide in S-Basal and arranged on agarose pad-lined glass microscopy slides for visualization. ImageJ software was used for quantification of fluorescence intensity. Background fluorescence was subtracted from the intestinal fluorescence and divided by worm size to generate a fluorescence intensity value. All photomicrographs show a collection of representative animals. For quantification, at least 20 worms were scored blindly in three independent replicates.

### Mitochondrial activity assays

#### Oxygen consumption rate (OCR) assay

The OCR assay was performed according to (Zuo et al., 2017) using the MitoXpress Xtra oxygen consumption assay kit (Agilent, USA). Approximately 100–150 worms were recovered from each bacterial diet plate and washed three times with S-basal solution to remove excess bacteria. The worms were then transferred to wells of a 96-well plate in a final sample volume of 90 μl. Then 10μl of the oxygen probe was added to each sample. The wells of the 96 well plate were then covered with two drops of mineral oil and immediately read using a Synergy Neo 2 plate reader using Gen5 software (BioTek, Wisnooski, VT, USA) in a time-resolved fluorescence mode with 380 nm excitation and 650 nm emission filters.

#### Measurement of ATP production

ATP was quantified using a bioluminescence ATP measurement kit (Thermo Fisher Scientific, Waltham, MA, USA). Worms were collected from NGM plates and washed three times in S-basal to remove bacteria and frozen at -80°C overnight. Before assessment, the samples heated to 95°C for 15 minutes and then cooled on ice for 5 minutes. The samples were then centrifuged at 14,000 x g for 10 min at 4°C and the supernatant used to measure ATP. 10 μl of each sample were transferred into 96-well plates in triplicates. The ATP assay solution was prepared according to the manufacturer’s instructions. 90 μl of the assay solution was then added to each sample. The sample wells were read on Synergy Neo 2 plate reader using Gen5 software (BioTek, Wisnooski, VT, USA) with a luminometer filter. An ATP standard curve was generated and the ATP concentration for each sample was calculated based on the standard curve.

#### Quantification of mitochondrial membrane potential

To assess mitochondrial membrane potential, worms were grown on NGM media seeded with particular *E*. *coli* diets containing 1.25 nM tetramethylrhodamine ethyl ester (TMRE) at 20°C for 3 days and visualized at the L4 stage.

#### Measurement of protein oxidation by OxyBlot

The OxyBlot protein oxidation detection kit (Millipore-Sigma, Burlington, MA, USA) was used to measure the level of protein oxidation. Worms were collected from each condition, washed with S-basal to remove bacteria and frozen to -80°C. After one hour, the samples were thawed and 100 μl of lysis buffer was added. Worms were homogenized using TissueLyser II (Qiagen, Germantown, MD, USA). The DNP reaction mixture was prepared by adding 35 μg protein for each sample adjusted in 7 μl, 3 μl of 15% SDS and 10 μl of DNP solution. The mixture was kept at room temperature for 15 min, then 7.5 μl of Neutralization buffer was added. The samples were then loaded and run in 10% SDS-PAGE gels. Next, they were transferred to nitrocellulose (Bio-Rad, Hercules, California, USA) and blocked with 5% non-fat milk for 1h. After washing with 1X PBS, the membrane was incubated with the first antibody (1:150) overnight at 4°C and then for 1h with the secondary antibody (1:300) at room temperature. Membranes were incubated with ECL plus detection reagent (Bio-Rad) and scanned using Chemiluminescent scanner (Bio-Rad). Band densities in a given lane were analyzed using ImageJ and added together as previously described [38]. Afterwards, the membranes were incubated with 15% hydrogen peroxide for 30 min at room temperature and treated with actin antibody. OxyBlot values were then normalized to actin for each sample.

### Development and fertility assays

Worm development was assessed by first synchronizing animals at the L1 stage and then quantifying developmental stage each day for 3 days. Approximately 100 animals were used for this assay. Developmental stage was scored based on vulva development stage. For the fertility assay, animals at the L4 stage were transferred to fresh seeded plates daily and the number of progeny on each plate counted.

### Thrashing assay

Worms were grown on NGM agar plates seeded with each *E*. *coli* bacterial strain until they reached the L4 stage of development. On days 1, 4, 8 and 12 the rate of animal movement was measured by quantifying their thrashing rate. Individual worms were placed in a 10 μl drop of S-basal on a microscope slide. After one minute of acclimation, the number of bends within 10 seconds were counted for each worm (with a total of 10 worms per experiment) blindly for a total of three biological replicates. One body bend was recorded as one rightward and one leftward body bend. The data was represented as number of thrashes per minute.

### Pumping rate

Pharyngeal contractions were recorded for each animal under high magnification using a Zeiss Observer Z1 microscope. Worms were transferred onto a new plate and allowed to acclimate for a few minutes. Pharyngeal pumping was then counted for 30 seconds for each worm for a total of three biological replicates. The data was represented as pumps per minute.

### Vitamin B12 restriction protocol

*E*. *coli* OP50 was grown in M9 medium at 37°C for 3 days. The bacteria were inoculated every 3 days into fresh M9 medium to be used as a food source for *C. elegans* [22].

To prepare B_12_-deficient worms, two to three L4 worms from the control plate were transferred onto plates containing B_12_-deficient M9 medium seeded with B_12_-deficient *E*. *coli*. Worms were grown on B_12_-deficient media for four generations until analysis [22].

### Lifespan analysis

All lifespan experiments were performed at 20°C. One hundred animals at the L4 stage were maintained on the *E*. *coli* strain for the duration of the assay and transferred every 1–2 days until animals no longer produced progeny. Animals were considered dead if they did not respond to touch using the platinum wire. Worms were censored if they escaped the plate or if they ruptured at the uterus. For supplementation of methylcobalamin or adenosylcobalamin, each metabolite was spread over the bacterial lawn to a final concentration of 0.2 μg/ml. Methionine and other amino acids were added to NGM media to a final concentration of 10μM. Doxycycline was added at a concentration of 6 μg/ml. GraphPad Prism version 8 (GraphPad Software, San Diego, California, USA) was used to calculate statistical significance where p-values were generated by the log-rank (Mantel-Cox) test. Statistical analysis was performed as previously described [39]. In order to achieve sufficient statistical power, three biological replicates were used for each lifespan experiment starting with 100 animals per plate. For each experiment where there were more than two strains, the log-rank test was performed on each strain individually compared to the control strain. Only experiments in which all three biological replicates showed identical statistics were considered. In all cases, p-values <0.05 are considered significant. Each lifespan figure represents one lifespan experiment. All lifespan trials and their statistics are provided in S1 Table.

### *C*. *elegans* pathogen infection assays

Worms were age-matched at the L4 stage by harvesting eggs using bleach/NaOH treatment of gravid hermaphrodites. Eggs were aliquoted on NGM plates containing *E*. *coli* (OP50, HT115, HB101, or BW25113) and then 50 L4 worms were transferred to *P*. *aeruginosa* PA14 infection plates. To prepare infection plates, PA14 was inoculated from a fresh culture plate and grown overnight at 37°C. The following day 15 μl of PA14 overnight culture was spotted onto NGM media plates. Plates were incubated overnight at room temperature and then transferred to 37°C for an overnight incubation and used for the survival assay the following day. Animal deaths were recorded daily every 2 hrs for a 12 hr period each day. Statistical analysis was performed as described for the lifespan assays.

To assess the level of infection, we grew wild-type and *mrpl-2(osa3)* animals on the various *E*. *coli* diets until the L4 stage at which time animals were transferred to plates containing *P*. *aeruginosa* expressing RFP. Infection levels were then measured based on the degree of fluorescence emitted in the worm gut lumen.

### Determination of vitamin B12 levels

Vitamin B12 levels were obtained using the Vitamin B12 ELISA Kit (Biovision). Around 2000 synchronized worms per biological replicate were grown on *E*. *coli* OP50 and HT115 plates, washed in S-basal, harvested, and homogenized in lysis buffer. Supernatants were used for determining the vitamin B12 content in samples following manufacturer’s instructions.

### Determination of methionine levels

Methionine content was determined using the Methionine Fluorescence Assay Kit (Abcam) and each experiment was performed in three biological replicates. Around 500 worms were grown on *E*. *coli* OP50 and HT115 plates, washed three times in S-basal, and then in 100 μl of Methionine assay buffer. Supernatants were used for determining the methionine content according to the manufacturer’s instructions.

### RNA sequencing analysis

Trizol extraction method was used to recover total RNA from worms and RNA was purified using Direct-zol RNA Kit (Zymo Research, CA, USA). An Agilent 2100 Bioanalyzer was used to assess RNA integrity. Library construction and sequencing was done by Novogene Inc. (CA, USA). In short, mRNA was enriched using oligo(dT) beads and rRNA removed using the Ribo-Zero kit. mRNA was fragmented, followed by synthesis of first and second strand cDNA synthesis. Then sequencing adapters were ligated and the double-stranded cDNA library completed through size selection and PCR enrichment. The library was sequenced using an Illumina Hiseq 4000 following manufacturer’s instructions for paired-end 150-bp reads. The raw data was cleaned by removing adapter sequences, reads containing poly-N and low-quality reads (Q<30) using Trimmomatic [40]. Tophat v.2.0.9 [41] was used to align clean reads to the *C*. *elegans* reference genome. The mapped reads from each sample was assembled using Cufflinks v.2.1.1 [41]. HTSeq v.0.6.1 [42] was used to count the number of reads mapped to each gene. In addition, the reads per kilobase million (RPKM) of each gene was calculated based on the length of the gene and the number of reads mapped to it. Differential expression analysis was performed using DESeq2 R package (v.1.10.1) [43]. Relative expression of genes with Benjamini-Hochberg corrected *P*-values (*Padj*)<0.05 were considered to be differentially expressed. Heatmaps were generated with pheatmap in R Studio.

## Supporting information

S1 Fig *atfs-1* RNAi impairs development/fertility of *sucg-1(osa2)* and *pdr-1*(*osa5)* but not *mrpl-2(osa3)* or *osa4* animals. (A) Photomicrographs and quantifications of developmental stage and fertility of wild-type, *sucg-1*(*osa2)*, *mrpl-2(osa3)*, *osa4*, or *pdr-1(osa5)* animals grown on control or *atfs-1* RNAi plates. (mean ±SD; *n* ≥ 20); ns denotes not significant, *** denotes p ≤ 0.001, **** denotes p ≤ 0.0001 (Student’s *t* test). (B) Photomicrographs and quantification of *hsp-6p*::GFP expression in wild-type, *sucg-1(osa2)*, *mrpl-2(osa3)*, *osa4*, or *pdr-1(osa5)* animals grown on control or *atfs-1* RNAi plates. Quantification of fluorescence intensities expressed as arbitrary units (A.U.); (mean ±SD; *n* ≥ 20); ns denotes not significant, **** denotes p ≤ 0.0001 (Student’s *t* test).
(TIF)

S2 Fig Germline rescue of *sucg-1(osa2)*, *mrpl-2(osa3)* and *pdr-1(osa5)*. (A) Photomicrographs of *hsp-6p*::GFP expression in wild-type, *sucg-1(osa2)*, and *sucg-1(osa2)* [*sucg-1*+]. (B) Photomicrographs of *hsp-6p*::GFP expression in wild-type, *mrpl-2(osa3)*, and *mrpl-2(osa3)* [*mrpl-2*+]. (C) Photomicrographs of *hsp-6p*::GFP expression in wild-type, wild-type [*pdr-1*+], *pdr-1(osa5)* [*pdr-1*+], and wild-type [*pdr-1*(*osa5*)].
(EPS)

S3 Fig Representative image of the OxyBlot experiment performed for Fig 2C. OxyBlots were generated as described in the Materials and Methods section. Arrows indicate density peaks that were used in the generation of the OxyBlot value.
(EPS)

S4 Fig No change in the developmental rates of *mrpl-2(osa3)* animals fed various *E*. *coli* diets. (A) Photomicrographs of wild-type and *mrpl-2(osa3)* animals fed *E*. *coli* OP50, HT115, HB101, and BW25113 and stained with TMRE dye. (B) Quantification of developmental stages at various time points for wild-type or *mrpl-2(osa3)* animals fed various *E*. *coli* diets (*n* = 3).
(EPS)

S5 Fig Rescue of *mrpl-2(osa3)* lifespan extension on a diet of *E*. *coli* OP50 through introduction of a wild-type *mrpl-2* transgene. (A, B) Lifespans of wild-type, *mrpl-2(osa3)*, *and mrpl-2(osa3)*[*mrpl-2*+] fed a diet of *E*. *coli* OP50. Independent transgenic line 1 (A) and line 2 (B) are shown.
(EPS)

S6 Fig Mild impairment to mitochondrial translation using doxycycline activates the UPR^mt^ and extends wild-type animal lifespan in a diet-dependent manner. (A) Quantification of *hsp-6p*::GFP expression in wild-type animals fed diets of *E*. *coli* OP50, HT115, HB101, or BW25113 in the presence or absence of 6 μg/ml doxycycline. Quantification of fluorescence intensities expressed as arbitrary units (A.U.); (mean ±SD; *n* ≥ 20); ns denotes not significant, *** denotes p ≤ 0.001 (Student’s *t* test). (B-E) Lifespans of wild-type animals fed diets of *E*. *coli* OP50, HT115, HB101, or BW25113 in the presence or absence of 6 μg/ml doxycycline.
(EPS)

S7 Fig Vitamin B12 levels are lower in animals fed *E*. *coli* OP50 relative to *E*. *coli* HT115. Quantification of vitamin B12 levels in wild-type animals fed *E*. *coli* OP50 versus HT115 (see Materials and Methods for details). (mean ±SD; *n =* 3); * denotes p ≤ 0.05 (Student’s *t* test).
(EPS)

S8 Fig Methionine levels are reduced in animals fed *E*. *coli* OP50 relative to those fed *E*. *coli* HT115. Quantification of methionine levels in wild-type animals fed *E*. *coli* OP50 versus HT115 (see Materials and Methods for details). (mean ±SD; *n =* 4); *** denotes p ≤ 0.001 (Student’s *t* test).
(EPS)

S9 Fig Methionine supplementation suppresses UPR^mt^ activation in *mrpl-2(osa3)* animals fed a diet of *E*. *coli* OP50. (A, B) Photomicrographs and quantification of *hsp-6p*::GFP expression of wild-type and *mrpl-2(osa3)* fed an *E*. *coli* OP50 diet in the presence or absence of the indicated amino acids at a concentration of 10 mM. Quantification of fluorescence intensities expressed as arbitrary units (A.U.); (mean ±SD; *n* ≥ 20); ns denotes not significant, * denotes p ≤ 0.05, ** denotes p ≤ 0.01, *** denotes p ≤ 0.001, **** denotes p ≤ 0.0001 (Student’s *t* test).
(TIF)

S1 Table Summary of lifespan and survival data with statistics. (XLSX)

S2 Table Summary of RNA sequencing analysis comparing wild-type, *mrpl-2(osa3)*, and *metr-1(ok521)* animals fed *E*. *coli* OP50. (XLSX)
