# Supplementary figures and images for: A novel gene-diet interaction promotes organismal lifespan and host protection during infection via the mitochondrial UPR
Source: PLoS Genet. 2020 Dec 18;16(12):e1009234. doi: 10.1371/journal.pgen.1009234 (PMC7781476; doi:10.1371/journal.pgen.1009234)

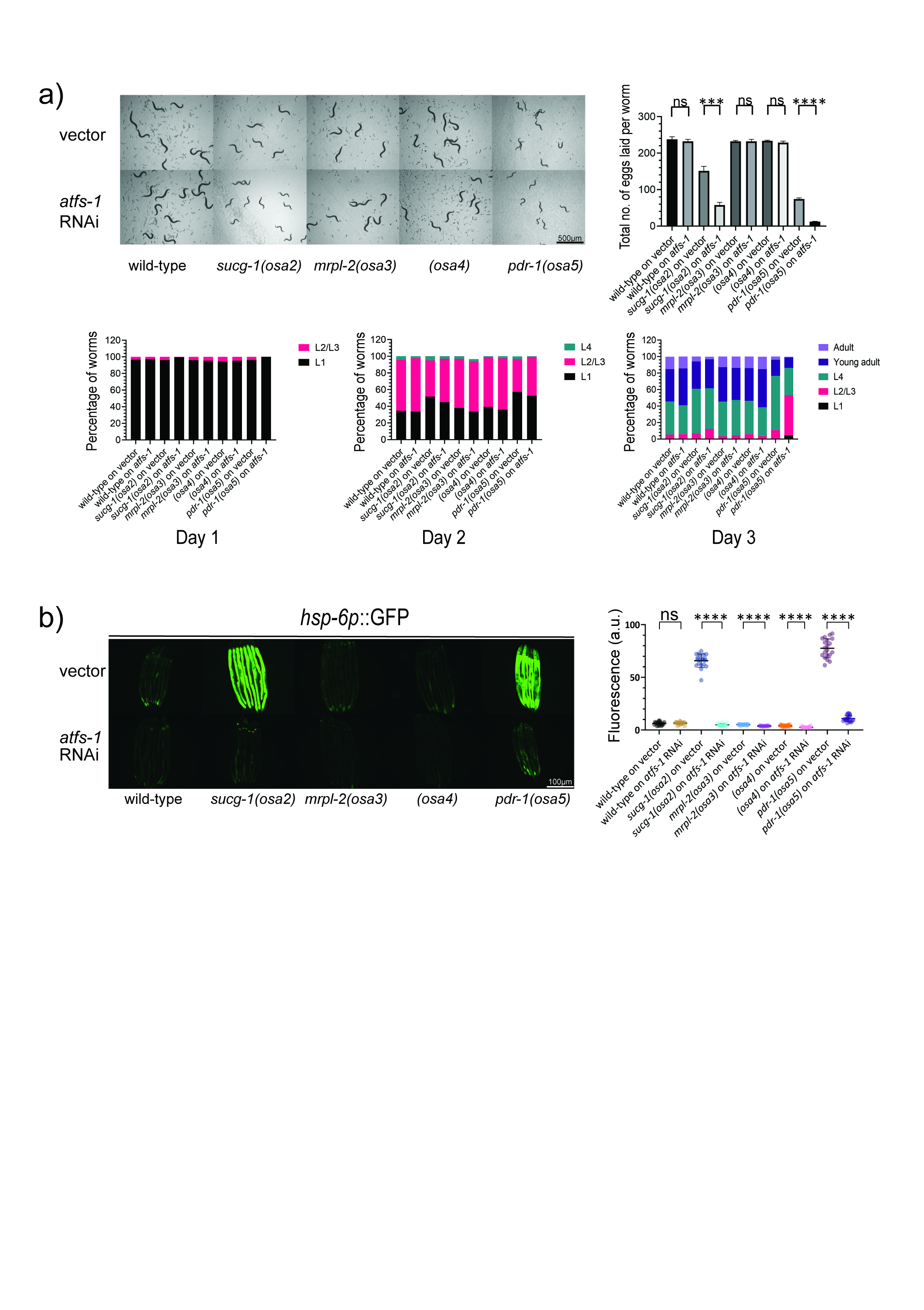

Supplement: S1 Fig — (A) Photomicrographs and quantifications of developmental stage and fertility of wild-type, sucg-1(osa2), mrpl-2(osa3), osa4, or pdr-1(osa5) animals grown on control or atfs-1 RNAi plates. (mean ±SD; n ≥ 20); ns denotes not significant, *** denotes p ≤ 0.001, **** denotes p ≤ 0.0001 (Student’s t test). (B) Photomicrographs and quantification of hsp-6p::GFP expression in wild-type, sucg-1(osa2), mrpl-2(osa3), osa4, or pdr-1(osa5) animals grown on control or atfs-1 RNAi plates. Quantification of fluorescence intensities expressed as arbitrary units (A.U.); (mean ±SD; n ≥ 20); ns denotes not significant, **** denotes p ≤ 0.0001 (Student’s t test). (TIF) [file pgen.1009234.s001.tif]

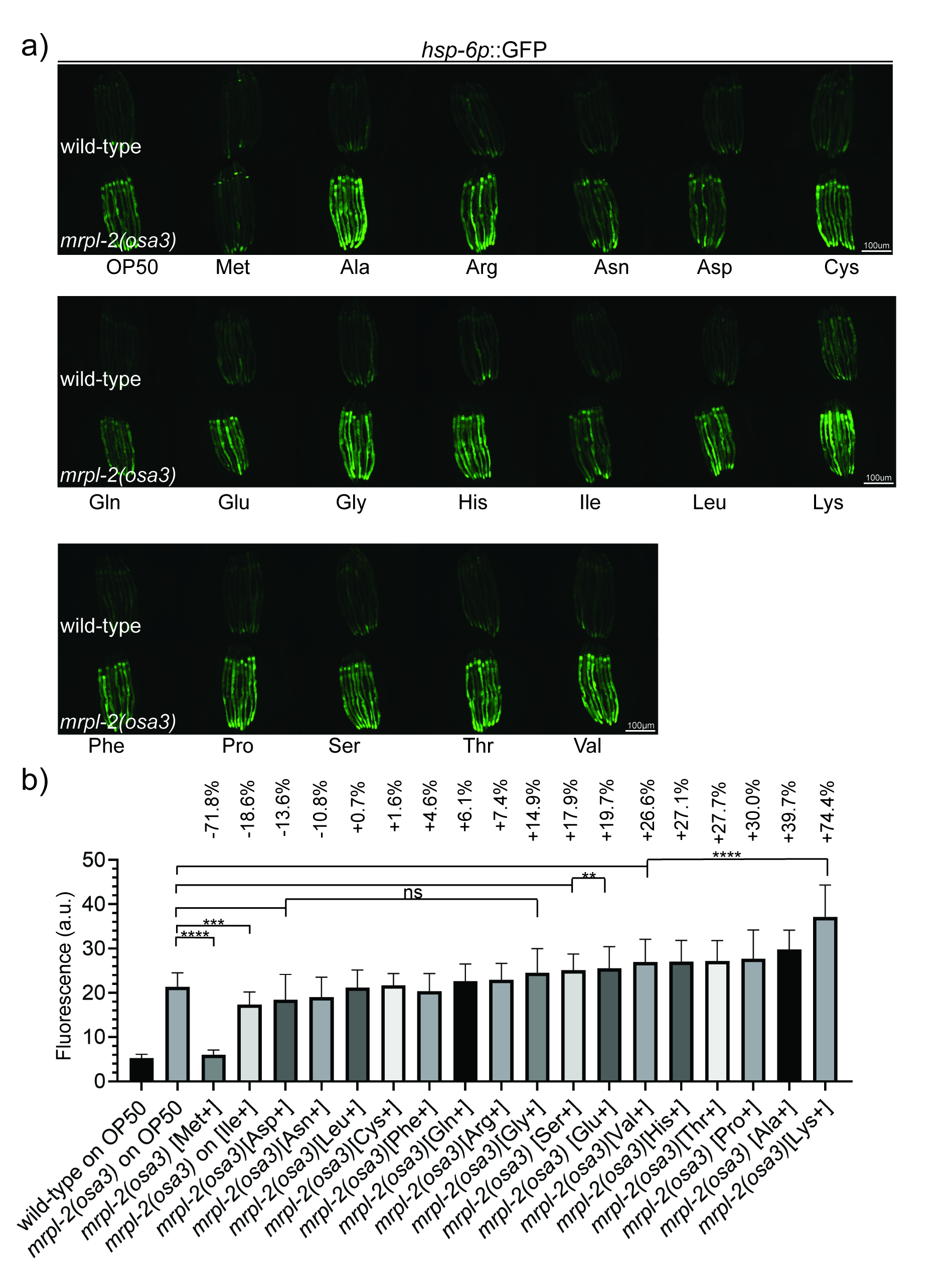

Supplement: S9 Fig — (A, B) Photomicrographs and quantification of hsp-6p::GFP expression of wild-type and mrpl-2(osa3) fed an E. coli OP50 diet in the presence or absence of the indicated amino acids at a concentration of 10 mM. Quantification of fluorescence intensities expressed as arbitrary units (A.U.); (mean ±SD; n ≥ 20); ns denotes not significant, * denotes p ≤ 0.05, ** denotes p ≤ 0.01, *** denotes p ≤ 0.001, **** denotes p ≤ 0.0001 (Student’s t test). (TIF) [file pgen.1009234.s009.tif]
